# Changes in TSH, T4, T3 and Thyroglobulin Levels throughout Total Thyroidectomy

**DOI:** 10.3390/jcm11092416

**Published:** 2022-04-25

**Authors:** Nora Senese, Jérôme R. Lechien, Kris Poppe, Alexandra Rodriguez, Didier Dequanter

**Affiliations:** 1 Department of Otolaryngology-Head & Neck surgery, CHU Saint Pierre, Université Libre de Bruxelles (ULB), 1000 Brussels, Belgium; nora.senese@stpierre-bru.be (N.S.); jerome.lechien@stpierre-bru.be (J.R.L.); alexandra.rodriguez@stpierre-bru.be (A.R.); 2 Department of Thoracic-Head & Neck Surgery, Centre Oscar Lambret, 59000 Lille, France; 3 Laboratory of Human Anatomy and Experimental Oncology, Faculty of Medicine and Pharmacy, Research Institute for Health Sciences and Technology, University of Mons (UMONS), 7000 Mons, Belgium; 4 Department of Endocrinology, CHU Saint Pierre, Université Libre de Bruxelles (ULB), 1000 Brussels, Belgium; kris.poppe@stpierre-bru.be; 5 Department of Stomatology-Maxillofacial Surgery, CHU Saint Pierre, Université Libre de Bruxelles (ULB), 1000 Brussels, Belgium

**Keywords:** thyroidectomy, hormonal release, thyrotoxicosis, Graves’ disease

## Abstract

Introduction: To assess the impact of total thyroidectomy on thyroid function. Methods: Monocentric observational prospective study. Patients who benefited from a total thyroidectomy in the Department of Otolaryngology-Head and Neck Surgery between September 2017 and July 2019 were included. Three blood samples were taken from each patient during the perioperative period: preoperatively (T_0_), intraoperatively (T_1_), and postoperatively (T_2_). Changes in TSH, T4, T3, and thyroglobulin levels were evaluated from T_0_ to T_2_. Epidemiological data were retrieved from the medical charts. Statistical analyses were performed for the entire cohort and subgroups regarding preoperative treatment and type of disease. Results: Seventy-seven patients were included in the study. T4 and thyroglobulin levels increased significantly from T_0_ to T_1_. TSH, T4, T3, and thyroglobulin levels decreased significantly from T_1_ to T_2_. Conclusions: Our study confirmed the hypothesis of variable kinetics of thyroid hormone levels associated with the manipulation of the thyroid gland during surgery, but none of these changes resulted in clinical effects, including thyrotoxicosis.

## 1. Introduction

Conventional thyroidectomy is a well-known and efficient surgery [1]. Over the past 20 years, it has undergone several improvements thanks to the development of new tools and techniques (bipolar thermofusion and intraoperative neuromonitoring, for example) and the specialization of surgical teams [2]. The indications for total thyroidectomy are also better selected based on the application of the ETA guidelines for the risks of ultrasound malignancy of thyroid nodules and the central role of the fine needle aspiration and the Bethesda system [3]. As a result of this development, surgical risks are increasingly limited. However, there is still a gray area regarding the risk of thyrotoxicosis crisis that would be triggered by thyroid surgery. The exact link between thyroidectomy and thyrotoxicosis crisis has not yet been demonstrated [4]. Moreover, the hormonal release caused by manipulating the thyroid gland during surgery remains hypothetical and has never been investigated yet. Guidelines support a euthyroid state before surgery to avoid perioperative complications related to hormone release [5,6]. These recommendations are based on the hypothesis that thyroid manipulation during surgery would cause a hormonal release into the blood circulation and might lead to a thyrotoxicosis crisis, in particular for Graves’ disease patients [7,8]. Thyrotoxicosis crisis is characterized by hyperthermia, heart failure, and digestive and central nervous system dysfunctions. The mortality can reach 10% due to multiple organ failure and congestive heart failure [9]. This study aims to assess the impact of total thyroidectomy on thyroid function.

## 2. Materials and Methods

The study protocol was approved by the Ethics Committee of CHU Saint-Pierre, Brussels (reference CE/17-09-06). Patients were invited to participate and were included in the study after their informed consent was obtained.

### 2.1. Study Design

From September 2017 to July 2019, every patient from the Department of Otolaryngology-Head & Neck Surgery of CHU Saint-Pierre, Brussels, Belgium, who benefited from a total thyroidectomy for any indication was included in this study.

Patients with the following conditions were excluded: severe coagulation disorders, pituitary dysfunction, and dementia limiting understanding of the study. Patients who underwent total thyroidectomy during total laryngectomy or partial pharyngolaryngectomy were also excluded.

Clinical and radiological characteristics were collected by reviewing the patients’ medical charts: age, sex, anti-thyroid drugs, thyroid hormone therapy, Graves’ disease, and thyroid lobes volume with ultrasound. Graves’ disease was detected by the demonstration of anti-thyroid stimulating hormone receptor antibodies, and a hyperthyroidism status was confirmed by ultrasound and scintigraphy. 

Three blood samples were taken from each patient during the perioperative period: on the induction of the anesthesia on the operating table, the day of the surgery (preoperatively = T_0_); 30 min after the removal of the thyroid gland, during the closure of the operating site (intraoperatively = T_1_); and the morning after surgery, at six o’clock (postoperatively = T_2_).

The anesthesia protocol was the same for every patient, including a preparation with Dexamethasone. The general anesthesia was obtained by target-controlled infusion (TCI) intravenously of Propofol and Remifentanil. 

The concentrations of serum thyrotropin (TSH), free thyroxine (T4), triiodothyronine (T3), and thyroglobulin were assessed. The laboratory tests were performed with the Chemiluminescence Centaur XP Siemens immunoanalyzer (Siemens Healthcare Diagnostics, Munich, Germany). The normal thyroid hormones and thyroglobulin concentration ranges were defined as TSH: 0.27–4.20 mU/L, T4: 12.0–22.2 pmol/L, T3: 3.10–6.80 pmol/L, and thyroglobulin: <77 µg/L.

According to our department’s protocol, one week after surgery, patients started hormone replacement therapy based on weight with a blood test six weeks later.

### 2.2. Statistical Analyses

Statistics were performed with the Statistical Package for the Social Sciences for Windows (SPSS version 25; IBM Corp, Armonk, NY, USA). The distribution of TSH, T4, T3, and thyroglobulin concentrations was assessed using the Kolmogorov–Smirnov test. Changes in pre- (T_0_) to post-surgery (T_2_) concentrations were evaluated by the Student’s *t*-test and the ANOVA with repeated measures tests for the parametric values and the Wilcoxon’s signed-rank and Friedman tests for the nonparametric values. First, analyses were made for the entire cohort. Thereafter, the cohort was divided into three subgroups. The first subgroup included patients who had not taken any treatment influencing thyroid function before surgery (i.e., antithyroid drugs, thyroid hormone therapy, and Lugol’s solution). The second subgroup included patients suffering from Graves’ disease. The last included the remaining patients (for example, patients with multinodular goiter taking thyroid hormone therapy). This third subgroup was not included in any further analysis. A *p*-value < 0.05 was considered statistically significant.

## 3. Results

### 3.1. Patient Demographics

Seventy-seven patients were included in the study. The majority of patients were female (*n* = 53). The mean age was 51 years (range 24–77 years). Total thyroidectomy was principally indicated to treat a multinodular goiter (Table 1). The mean volume of the thyroid lobes was 19 cc (2–133 cc) for the left side and 26 cc (3–183 cc) for the right side.

The administered preoperative treatments to the patients are listed in Table 1. All the patients with Graves’ disease were prepared with Lugol’s solution and antithyroid drugs. 

The cohort was divided into three subgroups regarding preoperative treatment and type of disease. A total of 50 patients who did not take any treatment influencing thyroid function before surgery (i.e., antithyroid drugs, thyroid hormone therapy, and Lugol’s solution) formed subgroup I (6 suspicions of malignancy, 1 medullary thyroid cancer, and 43 multinodular goiters). A total of 17 patients suffering from Graves’ disease formed subgroup II. The 10 remaining patients formed subgroup III.

Surgery had a significant impact on TSH, T4, T3, and thyroglobulin levels (Table 2). Significant differences between the mean levels at the three times of surgery (T_0_, T_1_, and T_2_) were observed (*p* < 0.001). Surgery produced a significant increase in T4 and thyroglobulin levels intraoperatively. Furthermore, surgery led to a significant decrease in TSH, T4, T3, and thyroglobulin levels postoperatively.

### 3.2. Subgroup Analysis

In the subgroups, surgery also had a significant impact on TSH, T4, T3, and thyroglobulin levels (Table 3 and Table 4). Significant differences between the mean levels at the three times of surgery (T_0_, T_1_, and T_2_) were observed (*p* < 0.001 and *p* < 0.05).

In the first subgroup (*n* = 50), surgery produced a significant increase in T4 and thyroglobulin levels intraoperatively. Then, significant decreases in TSH, T4, T3, and thyroglobulin levels were observed postoperatively (Table 3).

In the second subgroup with a more limited number of patients (*n* = 17), manipulation of the thyroid gland resulted in a significant increase in T4 and thyroglobulin levels and a significant decrease in T3 levels. 

Postoperatively, there were significant decreases in TSH, T4, and T3 levels, while a significant increase in thyroglobulin was observed (Table 4).

## 4. Discussion

Our study confirmed the unproven hypothesis that manipulation of the thyroid leads to changes in thyroid hormone levels. Indeed, we assessed significant changes in TSH, T4, T3, and thyroglobulin levels before and after total thyroidectomy. To our best knowledge, this is the first study to document the biological variations of the thyroid hormones throughout the perioperative period systematically.

Initially, we aimed to evaluate if the thyroid manipulation during total thyroidectomy did not lead to an important release of thyroid hormones as suspected in the literature [7,8,10]. Our study showed a moderate but significant increase in T4 and thyroglobulin levels close to normal limits. However, after surgery, we observed an important systematic decrease in all thyroid function tests, up to an extreme mean T3 concentration of 2.96 pmol.

In order to answer the second question, we evaluated the thyroid function tests fluctuations in the different subgroups. In our study, in the first subgroup (*n* = 50), surgery produced a significant increase in T4 and thyroglobulin levels intraoperatively. Then, significant decreases in TSH, T4, T3, and thyroglobulin levels were observed postoperatively. 

In the second subgroup, the limited considered patients with Graves’ disease presented two discrepancies. The T3 levels decreased during the intra and postoperative period. One of the explanations could be the state of hypothyroidism in which the patients were preoperatively and the consumption of T3 in the periphery. Interestingly, thyroglobulin levels continued to increase in the postoperative period. One of the hypotheses could be the phenomenon associated with the action of antithyroid drugs, which inhibit the thyroperoxidase and lead to an accumulation of thyroglobulin in the gland. Moreover, the patients with Graves’ disease were hypothyroid on the day of surgery. Thyroid hormone levels remained below normal during all three periods. However, no case of thyrotoxicosis crisis was diagnosed. This implies that another mechanism triggers a thyrotoxicosis crisis, then a sudden and elevated release of hormones. Although the number of patients with Graves’s disease is limited, this subgroup does not appear to be at greater risk for hormone release during surgical treatment. 

The same observation was made in Graves’ patients in whom a euthyroid state has not been reached, despite preparation [4].

Al Jassim et al. included 67 hyperthyroid patients with Graves’ disease who underwent a total thyroidectomy. In their study, no patient developed a thyroid storm. The preparation was variable and included: antithyroid drugs, beta-blockers, potassium iodine solution, and steroids. In our study, patients were also prepared with potassium iodine solution, although its clinical utility to reduce vascularity and friability of the gland remains unclear. However, some authors recommended its use based on its action in the Wolff–Chaikoff effect, which decreases the thyroperoxidase action in the presence of iodine and then decreases the synthesis and release of newly produced hormones [6]. 

In the literature, few studies have focused on the changes in the release of thyroid hormones during or after surgical treatment. In their study, Date et al. [11] determined in 10 patients during operation and the subsequent 18 days the concentrations of serum thyroid hormones and thyroglobulin (Tg). Serum Tg responded drastically, increasing from a low preoperative value (0.30 nmol/L) to a higher peak value (26 nmol/L) during the operation, followed by a gradual decline to levels lower than before surgery on day 18. Serum-free thyroxine T4 levels fluctuated only slightly during operation. Postsurgically, the values decreased to below 50% of the preoperative level. T3 concentration decreased during operation, whereafter the concentration increased slightly (day 2). The value of serum thyroid-stimulating hormone (TSH) decreased during and after the operation, but from day 10, the concentration began to rise steadily. Clearly, we observed in our study other kinetics and fluctuations in thyroid hormones even if the measurement times were different between the two studies. On the other hand, the conclusion is identical, namely the absence of clinical effects.

In their study, Shigemasa et al. [12] evaluated the sequential changes in serum thyroglobulin, free thyroxine, triiodothyronine, and thyrotropin in only ten patients, on whom thyroidectomy had been performed. Interestingly, these changes were compared with those in ten patients who underwent upper abdominal surgery. Marked elevations in serum Tg were observed after thyroid incision. On the other hand, the significant but minimal increases in serum FT4 and, similarly, serum T3 were observed at 24, 48, and 72 h after the thyroid incision. Interestingly, these increases were not observed during the upper abdominal surgery.

Lee et al. [13] demonstrated any change in the serum FT4 level and transient elevations of serum T3 after surgical treatment independently on the two groups of patients (*n* = 20) treated for two different pathologies. However, in our study, our preliminary results showed, in the different subgroups, a variability in sequential changes in serum thyroglobulin and thyroid hormones levels.

It is certain that additional studies are necessary in order to better clarify the preliminary observations, which, in comparison, with the few other studies, have an identical conclusion regarding the absence of clinical effects.

## 5. Conclusions

We have documented the biological changes and variations of thyroid hormones throughout total thyroidectomy. Our study confirmed the hypothesis of variable kinetics of thyroid hormone levels associated with manipulation of the thyroid gland during surgery. However, none of these changes resulted in clinical effects, including thyrotoxicosis. 

## Figures and Tables

**Table 1:** Indications for surgery, patient characteristics, and preoperative treatment.

Indication	Number	Preoperative Treatment				
		Levothyroxine	PTU **	Thiamazol	Lugol †	No Treatment Influencing Thyroid Function
Multinodular goiter	52	9	0	0	0	43
Graves’ disease	17	6	5	12	17	0
Suspicion of malignancy *	6	0	0	0	0	6
Medullary Thyroid Cancer	1	0	0	0	0	1
Toxic Adenoma	1	0	0	1	0	0
Total	77	15	5	13	17	50

* Suspicion of malignancy in fine-needle aspiration biopsy; ** Propylthiouracil; † Lugol’s solution: iodine 5 g, iodide 10 g, water ad 100 mL. 5 drops 3 times daily 10 days before the surgery. The doses of levothyroxine were between 25 and 100 µg per day, between 50 and 200 mg of PTU, and between 10 and 20 mg of Thiamazole.

**Table 2:** Mean levels and confidence intervals of TSH, T4, T3 and thyroglobulin perioperative in the entire cohort.

	T_0_	T_1_	T_2_	T_0_ vs. T_1_	T_0_ vs. T_2_	T_1_ vs. T_2_
TSH (mU/L)	2.77 (1.52–3.99)	2.86 (1.51–4.21)	1.42 (0.68–2.16)	*p* > 0.05	*p* < 0.001	*p* < 0.001
FT4 (pmol/L)	15.40 (14.50–16.30)	16.76 (15.79–17.73)	14.06 (13.12–15.00)	*p* < 0.001	*p* < 0.001	*p* < 0.001
FT3 (pmol/L)	4.71 (4.49–4.93)	4.68 (4.46–4.90)	2.96 (2.72–3.20)	*p * > 0.05	*p* < 0.001	*p* < 0.001
Thyroglobulin (µg/L)	777.59 (31.91–1523.27)	2147.23 (958.71–3335.75)	1122.15 (531.73–1712.57)	*p* < 0.001	*p* < 0.001	*p* < 0.001

The normal values set are TSH 0.27–4.20 mU/L, free T4 12.0–22.2 pmol/L, free T3 3.10–6.80 pmol/L, and thyroglobulin < 77 µg/L.

**Table 3:** Mean levels of TSH, T4, T3, and thyroglobulin perioperative in subgroup I (*n* = 50).

	T_0_	T_1_	T_2_	T_0_ vs. T_1_	T_0_ vs. T_2_	T_1_ vs. T_2_
TSH (mU/L)	1.27	1.62	0.78	*p = 0.02*	*p* < 0.001	*p* < 0.001
T4 (pmol/L)	16.71	18.23	15.31	*p* < 0.001	*p* < 0.001	*p* < 0.001
T3 (pmol/L)	4.88	4.95	3.15	*p * > 0.05	*p* < 0.001	*p* < 0.001
Thyroglobulin (µg/L)	446	2202	861	*p* < 0.001	*p* < 0.001	*p* < 0.001

The normal values set are TSH 0.27–4.20 mU/L, free T4 12.0–22.2 pmol/L, T3 3.10–6.80 pmol/L, and thyroglobulin < 77 µg/L.

**Table 4:** Mean levels of TSH, T4, T3, and thyroglobulin perioperative in subgroup II (*n* = 17).

	T_0_	T_1_	T_2_	T_0_ vs. T_1_	T_0_ vs. T_2_	T_1_ vs. T_2_
TSH (mU/L)	7.41	7.16	3.24	*p > 0.05*	*p = 0.01*	*p = 0.01*
T4 (pmol/L)	11.12	12.25	10.17	*p = 0.01*	*p* < 0.01	*p* < 0.001
T3 (pmol/L)	4.28	3.97	2.37	*p = 0.03*	*p* < 0.01	*p* < 0.001
Thyroglobulin (µg/L)	2109.48	2666.12	2962.09	*p = 0.03*	*p < 0.01*	*p > 0.05*

The normal values set are TSH 0.27–4.20 mU/L, free T4 12.0–22.2 pmol/L, T3 3.10–6.80 pmol/L, and thyroglobulin < 77 µg/L.

## Data Availability

The data presented in this study are available on request from the corresponding author. The data are not publicly available due to private reasons.
